# An Automated Solid-Phase Extraction–UPLC–MS/MS Method for Simultaneous Determination of Sulfonamide Antimicrobials in Environmental Water

**DOI:** 10.3390/molecules28124694

**Published:** 2023-06-11

**Authors:** Mengyu Qi, Pengfei He, Hongmei Hu, Tongtong Zhang, Tiejun Li, Xiaoning Zhang, Yilin Qin, Yingjie Zhu, Yuanming Guo

**Affiliations:** 1 Institute of Marine and Fisheries, Zhejiang Ocean University, Zhoushan 316021, China; 2 Key Laboratory of Sustainable Utilization of Technology Research for Fisheries Resources of Zhejiang Province, Zhejiang Marine Fisheries Research Institute, Zhoushan 316021, China; 3 Department of Chemistry, Zhejiang University, Hangzhou 310007, China; 4 State Key Laboratory of Resource Insects, College of Sericulture, Textile and Biomass Sciences, Southwest University, Chongqing 400715, China

**Keywords:** sulfonamide antimicrobials, automated solid-phase extraction, isotope dilution, ultra-high performance liquid chromatography–tandem mass spectrometry, environmental water

## Abstract

The large-scale use of sulfonamide antimicrobials in human and veterinary medicine has seriously endangered the ecological environment and human health. The objective of this study was to develop and validate a simple and robust method for the simultaneous determination of seventeen sulfonamides in water using ultra-high performance liquid chromatography–tandem mass spectrometry coupled with fully automated solid-phase extraction. Seventeen isotope-labeled internal standards for sulfonamides were used to correct matrix effects. Several parameters affecting extraction efficiency were systematically optimized, and the enrichment factors were up to 982−1033 and only requiring about 60 min per six samples. Under the optimized conditions, this method manifested good linearity (0.05–100 μg/L), high sensitivity (detection limits: 0.01–0.05 ng/L), and satisfactory recoveries (79–118%) with acceptable relative standard deviations (0.3–14.5%, *n* = 5). The developed method can be successfully utilized for the determination of 17 sulfonamides in pure water, tap water, river water, and seawater. In total, six and seven sulfonamides were detected in river water and seawater, respectively, with a total concentration of 8.157–29.676 ng/L and 1.683–36.955 ng/L, respectively, and sulfamethoxazole was the predominant congener.

## 1. Introduction

Sulfonamides (SAs), as a class of synthetic antimicrobials with a *p*-aminobenzene structure, have been widely used in human and veterinary medicine for the treatment of various bacterial, protozoal, and fungal infections [1,2]. Owing to their broad-spectrum activity, low price, and definite effects, SAs are very popular in the European Union and China [3]. In 2017, SAs and trimethoprim represented 2.85% of the antimicrobial consumption in the European Union/European Economic Area (EU/EEA) [4], while the use of SAs was as high as 5% in China [5]. However, SAs cannot be entirely metabolized in the body, leading to a large part being excreted through urine or feces [6]. Given that wastewater treatment plants (WWTPs) cannot eliminate them, substantial amounts of SAs could be released into the terrestrial and aquatic environments, posing a threat to human health and environmental ecosystems [7]. The occurrence of SAs in surface water and groundwater has been widely reported with concentrations ranging from nanograms to micrograms per liter [7]. Toxicological studies have shown that SAs can induce blood disorders, liver damage, cancer, and tumors, and environmental SAs can lead to the development of bacteria with antibiotic-resistant genes [8]. To prevent the potential risk, the European Union and China have set a maximum residue limit (MRL) of 100 μg/kg and an acceptable daily intake of 50 μg/kg for total SAs in foods of animal origin [9]. But until now, no MRLs have currently been set for SAs in environmental water. Thus, to protect public health and the aquatic environment, attempts have been made to develop novel methods for the highly efficient extraction and determination of trace SAs in various water matrices.

Compared to enzyme-linked immunosorbent assays (ELISAs) and biosensing analysis, chromatography-based techniques are more preferable for SA determination [9], such as high-performance liquid chromatography (HPLC) [10,11], liquid chromatography–tandem mass spectrometry (LC-MS/MS) [5,12,13,14], ultra-high performance liquid chromatography–tandem mass spectrometry (UPLC-MS/MS) [1,6,15,16], and capillary electrophoresis (CE) [17,18]. UPLC–MS/MS, in particular, has been widely recommended due to its superior sensitivity, selectivity, and fast analysis efficiency in recent years [19].

Due to the low concentration of SAs in real water samples and the co-existence of matrix impurities, sample pretreatment techniques including solid-phase extraction (SPE) [1,15,20], dispersive liquid–liquid microextraction (DLLME) [21], solid-phase microextraction (SPME) [13], and magnetic solid-phase extraction (MSPE) [6] have been utilized before chromatographic analysis. Although MSPE and SPME show advantages in separating or recycling the sorbent in a fast and simple manner, they need complicated operation procedures to synthesize the composite sorbent materials. DLLME is easier to implement rapidly and is environmentally friendly, but it suffers from poor sensitivity. SPE is one of the most widely used technologies in sample pretreatment due to its high sensitivity. Nevertheless, traditional SPE has obvious drawbacks, including the demand for a large volume of samples, expensive cartridges, and long sample pretreatment times, as well as multiple manual steps which raise the possibility for artificial errors and unstable recovery [22]. Recently, automated online SPE coupled with UPLC–MS/MS has been successfully applied in the determination of SA residues in environmental and treated waters, which dramatically reduced the sample volume (~10 mL) and extraction time (~20 min) [16]. However, the sensitivity, reliability, and repeatability were poor.

In this study, we aimed to establish a simple but effective analytical method for the determination of 17 SAs in various water matrices, based on UPLC–MS/MS coupled with an automated cartridge-disk universal SPE system (in-built Poly-Sery HLB SPE cartridge). It has been well documented that matrix effects from water samples can cause differences in the ionization of the analyte of interest in electrospray ionization tandem mass spectrometry analysis [19]. Attempts were made to correct the matrix effects with 17 isotope-labeled internal standards (ILISs) for SAs. After optimization and validation, the proposed method is expected to improve the determination of antimicrobial pollution in water.

## 2. Results and Discussion

### 2.1. Optimization of Automated Solid Phase Extraction Procedures

The extraction efficiency of automated SPE is influenced by many factors, such as sorbent type, elution solvent, eluent volume, Na_2_EDTA addition, ionic strength, and sample pH. To achieve the highest possible sensitivity, ultrapure water spiked with 20 ng/L of SAs was selected for the automated SPE recovery experiments.

#### 2.1.1. Sorbent Type

**Figure 1:**
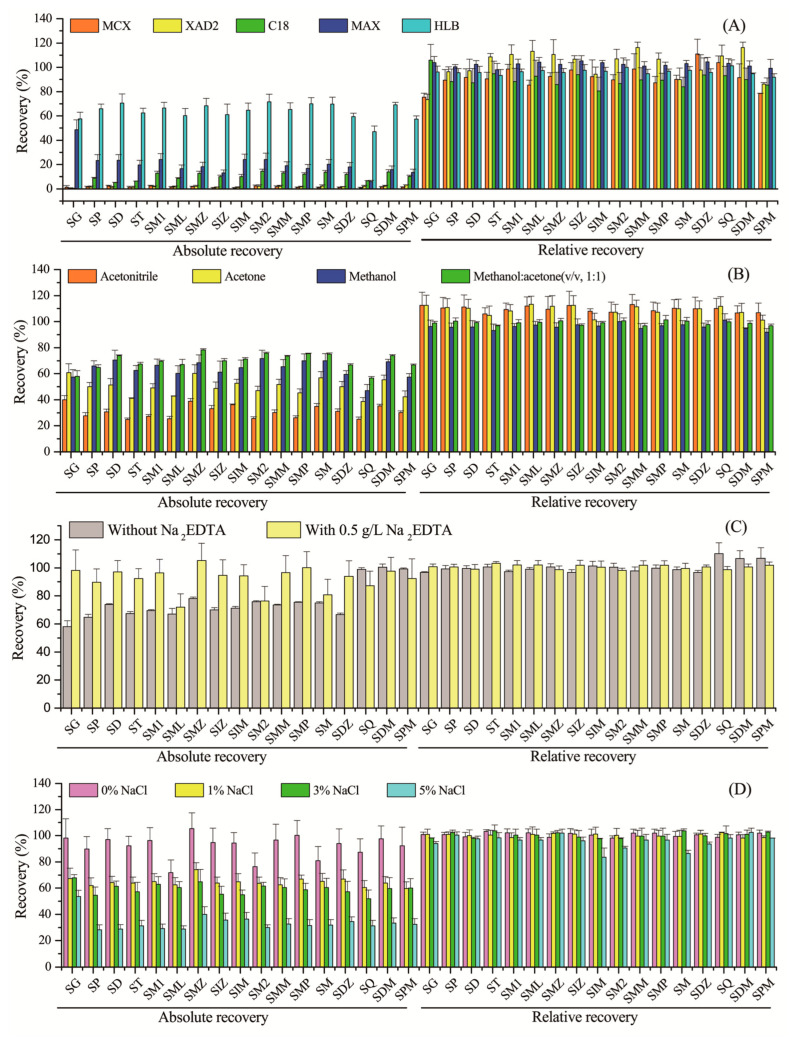
(**A**) Effect of cartridge sorbents (*n* = 3); (**B**) effect of eluents (*n* = 3); (**C**) effect of Na_2_EDTA addition (*n* = 3); (**D**) effect of ionic strength (*n* = 3).

During the method development, the five adsorbents listed in Table 1 were evaluated for extraction of the SAs from water using 8 mL of methanol as an elution solvent. Both the external standard method (absolute recovery) and the ILIS method (relative recovery) were used for calculating the extraction recovery (Figure 1A). The highest absolute recoveries were obtained using the CNW Poly-Sery HLB (range 47–72%, mean 64%), followed by CNW Poly-Sery MAX (6.0–49%, 20%), CNWBOND LC-C18 (0.47–14%, 10%), CNW Poly-Sery XAD2 (0.66–3.1%, 1.8%), and CNW Poly-Sery MCX (0.78–2.5%, 1.5%). As a result, CNW Poly-Sery HLB was chosen for the optimization.

Overall, good relative recoveries (73–116%) were observed for 17 SAs in all the SPE cartridges due to the same constitutional formula of the native analytes and their corresponding ILISs.

#### 2.1.2. Elution Solvent and Eluent Volume

The elution solvent is a critical factor that has a significant influence on the retention and adsorption capability of sorbents toward target compounds [23]. Figure 1B shows the effect of the four eluents on the elution of the extracted SAs from the selected cartridge. Methanol–acetone (*v*/*v*, 1:1) exhibited the best elution performance, with absolute recoveries ranging from 57% to 78% (mean 70%), which was slightly higher than those of methanol (mean 64%). The lower absolute recoveries were obtained using acetone (range 39–61%, mean 50%) and acetonitrile (25–40%, 31%). However, it is worth noting that the relative recoveries of 17 SAs ranged from 92% to 113% for three eluents. Hence, methanol–acetone (*v*/*v*, 1:1) was selected as the best elution solvent for the following study.

With the elution solvent fixed, the effect of eluent volume was studied by varying the volume from 3 to 10 mL, and no obvious change was observed when the volume was over 8 mL. As a result, 8 mL of acetone–methanol (*v*/*v*, 1:1) was sufficient to elute the SAs from the selected cartridge (Supplementary Materials, Figure S1).

#### 2.1.3. Effect of Na_2_EDTA

The metal ions in real water samples may form antibiotic–metal complexes (AMCs) with antibiotics, which affects the extraction efficiency. Antibiotics with more electron-rich groups containing N and O may lead to a stronger complexation with metal ions [24]. The structures of SAs contain both amino nitrogen and amide nitrogen, which may interact with heavy metals such as Fe^2+^, Co^2+^, Cu^2+^, and Ni^2+^, to form AMCs [25]. According to the previous work and sample matrix [20], 0.5 g/L of Na_2_EDTA was used to reduce metal ion interference in this study. Satisfactorily, the addition of Na_2_EDTA enhanced the signal response. The absolute recoveries with Na_2_EDTA addition (range 72–105%, mean 92%) were higher than those without Na_2_EDTA addition (57–78%, 70%) (Figure 1C). Meanwhile, the relative recoveries ranged from 97% to 103% with or without Na_2_EDTA, which met the accuracy requirements of the method.

#### 2.1.4. Effect of Ionic Strength and pH Value

To assess the applicability of the method to fresh and seawater, the effect of ionic strength was investigated. As shown in Figure 1D, the extraction efficiency was decreased with the increase of NaCl concentration from 0% (absolute recovery range 72–105%, mean 92%) to 5% (28–54%, 33%). However, relative recoveries (range 84–104%) were similar in different ionic strengths. The salinity of natural seawater is about 35 [19], and that of natural fresh water is negligible. Hence, no salt addition was an optimal choice both for freshwater and seawater. 

Sample pH is expected to significantly influence the speciation of the target 17 SAs owing to the presence of one basic amine group (–NH_2_) and one acidic sulfonamide group (–SO_2_NH–) in their structures (Supplementary Materials, Figure S2). The pK_a1_ (1.52–2.90) and pK_a2_ (4.71–8.54) [26] shown in Table 2 indicate that protonation and deprotonation of these SAs occur readily under specific pH conditions. SAs are positively charged at pH 2, neutral between pH 2 and 5, and negatively charged at pH values above 5. The interaction with the cartridge material is stronger for analytes in neutral forms [27]. Typically, the sample pH was adjusted to a value of about 2.0–4.0 [27]. Therefore, the sample pH was fixed at 3.0 in this study.

### 2.2. Matrix Effect

**Figure 2:**
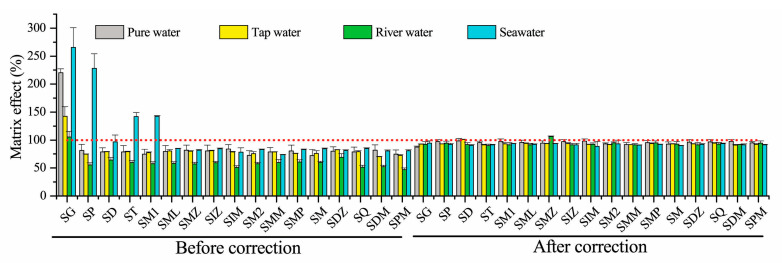
Matrix effects in 4 different water matrices.

Suppression or enhancement of analyte responses by co-extracted matrix components is commonly encountered when the instrument analysis is based on UPLC–MS/MS [28]. In this study, the matrix effects (ME) were evaluated by the determination of unspiked and spiked (20.0 μg/L) real water sample extracts. The calculation formula was ME (%) = (A_e_ − A_0_)/A_s_ × 100%, where A_e_, A_0_, and A_s_ were the signal intensity of the spiked extracts, unspiked extracts, and standard solution, respectively. When the ME value is greater than or less than 100%, the signal is enhanced or suppressed. The matrix effect existed in the tested matrices to some extent (Figure 2). The matrix effects for most of the SAs were in the range of 70–84% in pure water and tap water except SG (220% in pure water and 142% in tap water). Obvious signal enhancements were observed for SG (265%), SP (228%), ST (142%), and SM1 (143%) in seawater, while there was a slight signal suppression for the other 13 SAs (74–97%). Furthermore, in river water, 16 SAs showed signal suppression (47–69%), while SG showed slight signal enhancement (106%).

Notably, no matrix effect was observed for all SAs in the four matrices (ME range 88–106%) after ILIS calibration (Figure 2). Thus, the use of ILISs proved to be effective in correcting matrix effects without the need for further processing or the use of time-consuming matrix-matching calibration.

### 2.3. Evaluation of the Method Performance

The developed automated SPE–UPLC–MS/MS method was validated after assessing its performance based on evaluations of its linearity, limits of detection (LODs), limits of quantitation (LOQs), enrichment factors (EFs), and precisions (Table 3). The calibration curves of 17 SAs were established using the ILISs in concentrations of 0.05–100 μg/L. The linear relationship was good, and the correlation coefficient (r^2^) was in the range of 0.9992–0.9999. The LODs and LOQs ranged from 0.01–0.05 ng/L (S/N = 3) and 0.03–0.15 ng/L (S/N = 10), respectively. EFs were determined by calculating the ratio of the equilibrium concentration of analytes in the initial mobile phase to the original concentration of analytes in the aqueous phase, obtaining values of 982−1033 in this study. Intra- (*n* = 5) and inter-day (*n* = 5) precisions were calculated by extracting the analytes from ultrapure water samples at the level of 20 ng/L and relative standard deviations (RSDs) lower than 4% and 9% were obtained, respectively (Table 3). These results demonstrated a high sensitivity and excellent repeatability of the proposed method.

Furthermore, the accuracy of the method was evaluated by four different spiked concentrations of 17 SAs (1, 10, 20, and 100 ng/L) (Supplementary Materials, Table S1). It can be seen that in pure water, tap water, river water, and seawater, the recoveries of the SAs ranged from 79–105%, 101–117%, 90–104%, and 104–118%, respectively, with RSDs of 1.2–14.5%, 0.9–7.0%, 5.0–11.4%, and 0.3–5.1% (*n* = 5), respectively. As a result, the recovery and precision of this method were satisfactory, which could meet the requirements for the determination of SAs in real environmental water.

The proposed method was compared with the recently reported methods in terms of sensitivity, accuracy, precision, and sample pretreatment speed (Table S2). The results show that the LODs of the proposed method are comparable to manual SPE UPLC–MS/MS [15], but superior to those obtained with MSPE UPLC–MS/MS [6], MIP-SPE HPLC-PDA [10], online SPE HPLC–MS/MS [12], online SPE UPLC–MS/MS [16], and in situ derivatization and HF LPME UPLC-FLD [29], and much lower than in-tip SPME HPLC-PDA [11] and DLLME UPLC-DAD [21]. Nonetheless, compared to manual SPE, automated SPE can reduce the extraction time for the same sample volume. For 1000 mL water samples, the total run time of automated SPE was about 60 min/6 samples, while the manual SPE needed more than 250 min. Furthermore, the commercial CNW Poly-Sery HLB was used for the first time to extract SAs from water instead of the most commonly used Oasis HLB. However, the LODs, recoveries, and RSDs of this method were similar or better than those of reported methods, demonstrating that the proposed method is a rapid, sensitive, repeatable, and eco-friendly method for the analysis of SAs from various water samples.

### 2.4. Real Water Analysis

**Figure 3:**
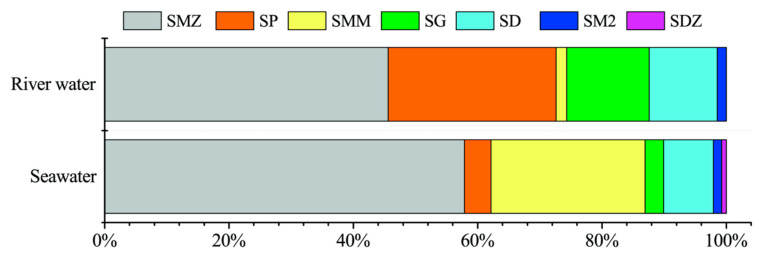
Distribution of SAs in river water and seawater.

To evaluate the applicability of the proposed method, four different water matrices were analyzed. No SAs was detected both in Wahaha pure water and tap water. As shown in Supplementary Materials, Table S3, six and seven SAs were detected in river water and seawater, respectively, with the total concentration of SAs (∑SAs) ranging from 8.157–29.676 ng/L (mean 17.701 ng/L) and 1.683–36.955 ng/L (16.984 ng/L), respectively. The distributions of SAs in river water and seawater were similar (Figure 3). SMZ was predominant in river water (46%) and seawater (58%), followed by SP (27%) in river water and SMM (25%) in seawater. The results are consistent with many previous studies [1,30,31,32]. For example, among the studied compounds, SMZ (max. 78.88 ng/L) and SP (max. 38.88 ng/L) were the most common pollutants identified in surface water in the most heavily urbanized area of Poland [1]. As described by Duan et al., the concentrations of SAs in North America (USA and Canada) were significantly lower than those in South Africa, and SMZ was determined to be the dominant pollutant in most countries [32]. A high concentration of SMZ was found owing to its widespread use in agriculture, aquaculture, and livestock because of its low cost. Rainwater runoff and riverine inputs will promote the occurrence of SAs in the different aquatic environments. For instance, mass flow calculations estimate that 12 tons of SMZ are discharged annually from the Mekong River into the South China Sea [31].

## 3. Materials and Methods

### 3.1. Chemicals and Reagents

Seventeen SAs standards, i.e., sulfaguanidine (SG), sulfapyridine (SP), sulfadiazine (SD), sulfathiazole (ST), sulfamerazine (SM1), sulfamethizole (SML), sulfamethoxazole (SMZ), sulfisoxazole (SIZ), sulfisomidine (SIM), sulfamethazine (SM2), sulfamonomethoxine (SMM), sulfamethoxypyridazine (SMP), sulfameter (SM), sulfachloropyridazine (SDZ), sulfaquinoxaline (SQ), sulfadoxine (SDM), and sulfadimethoxine (SPM) were purchased from ANPEL Laboratory Technologies (Shanghai, China). Their corresponding 17 ILISs, including SG-D_4_, SP-^13^C_6_, SD-^13^C_6_, ST-D_4_, SM1-^13^C_6_, SML-^13^C_6_, SMZ-^13^C_6_ SIZ-^13^C_6_, SIM-D_4_, SM2-D_4_, SMM-D_4_, SMP-D_3_, SM-D_4_, SDZ-^13^C_6_, SQ-^13^C_6_, SDM-D_3_, and SPM-D_6_, were purchased from Cato Research Chemicals Inc (Eugene, Oregon, USA), Dr. Ehrenstorfer GmbH (Augsburg, Germany), or Toronto Research Chemicals (Toronto, Canada). Methanol, acetone, acetonitrile, ethyl acetate, and formic acid of HPLC grade were provided by Merck (Darmstadt, Germany). Sodium chloride (NaCl) and disodium ethylenediamine tetraacetate (Na_2_EDTA) were obtained from Sinopharm Chemical Reagent Co (Beijing, China). Ultrapure water was prepared by a Milli-Q Plus 185 system (Millipore Corporation, Burlington, MA, USA). The stock standard solutions of 17 SAs and 17 ILISs (1 mg/L) were prepared in methanol and stored in the dark at −20 °C. Then, fresh calibration standard solutions were prepared daily by diluting the mixed standard solution with the initial mobile phase.

### 3.2. Sampling and Preparation

A total of 3 Wahaha pure water samples, 3 tap water samples, 6 river water samples, and 12 surface seawater samples were collected from a shop, a laboratory, Lincheng River in Zhoushan, and Sanmen Bay, East China Sea, respectively, in November 2021, and the sampling locations are shown in Figure S3. The collected river water or seawater samples were filtered through 0.22 μm microfiber filters to eliminate suspended solids and phytoplankton and then stored at 4 °C until extraction.

### 3.3. Automated Solid-Phase Extraction

An automated cartridge-disk universal SPE system (LabTech, Beijing, China) (Supplementary Materials, Figure S4), which can process six samples simultaneously, was used to extract the target SAs from water. To ensure the best extraction performance for all target compounds, five types of commercial SPE cartridges from CNW Technologies (Duesseldorf, Germany) were evaluated for extraction efficiency. The nature and properties of these cartridges are given in Table 1. 

The automated procedure was set as follows: 1.0 L of the filtered water sample spiked with 20 ng of a mixture of the 17 ILISs was loaded onto the cartridge which had been preconditioned with 8 mL of methanol and 8 mL of ultrapure water. After sample loading, the SPE cartridges were rinsed with 10 mL of acidified ultrapure water (pH 3.0) and then dried under N_2_ blowdown for 10 min. The SPE cartridges were eluted with 8 mL of acetone–methanol (*v*/*v*, 1:1). The whole procedure was completely automated. Then, the collected eluents were concentrated to dryness using a 45 positions N-EVAP/13165 nitrogen Evaporator from Organomation (Berlin, MA, USA) and re-dissolved in 1 mL of the initial mobile phase. This solution was filtered through a 0.22 μm filter before UPLC–MS/MS analysis.

### 3.4. Instrumental Analysis

The target SAs were analyzed by a Waters Acquity UPLC I-Class system (Waters, Milford, MA, USA) coupled with a Xevo TQ-S triple quadrupole mass spectrometer (Waters, Manchester, UK) in multiple reaction monitoring (MRM) modes. The target compounds were separated on a Waters BEH C18 column (2.1 mm × 100 mm, 1.7 μm) after 5 μL of sample extract was injected. The column temperature was maintained at 40 °C. The mobile phase comprised eluent A (2 mmol/L ammonium acetate solution containing 0.1% formic acid) and eluent B (acetonitrile). A gradient program was used for separation at a flow rate of 0.30 mL/min with 90% A (0 min), 90% A (1.5 min), 87.5% A (6.5 min), 70% A (9.5 min), 60% A (10.5 min), 10% A (10.7 min), 10% A (11.5 min), 90% A (11.8 min), and finally 90% A (15 min).

The MS/MS was operated in positive electrospray ionization (ESI+). Nitrogen (99.99%) and argon (99.9999%) were used as the desolvation gas and collision gas, respectively. The ESI+ operating conditions of the source were as follows: capillary voltage = 3.0 kV; desolvation temperature = 600 °C; source temperature, 150 °C; desolvation gas flow = 800 L/h; and cone gas flow = 150 L/h. Table 2 lists the MRM transition conditions of each compound, and their mass spectra are shown in the Supplementary Materials, Figure S5.

## 4. Conclusions

In summary, a facile and robust automated SPE procedure combined with UPLC–MS/MS was developed for the simultaneous and sensitive determination of 17 SAs in various water matrices. No significant matrix effects were observed after the correction with the corresponding ILISs. Compared with other methods found in the literature, the proposed optimized method was simpler, faster, and had higher sensitivity but yields reasonable recovery and good reproducibility, which is desirable for routine methods. Among the detected SAs, SMZ was predominant in river water and seawater, followed by SP in river water and SMM in seawater. Further investigation is required to evaluate the fate, behavior, and ecological and health risks of these compounds in environmental waters.

## Figures and Tables

**Table 1:** Nature and properties of tested SPE cartridges.

SPE Cartridge	Dimensions	Sorbent Properties
CNW Poly-Sery HLB	50–80 μm ^a^, 500 mg ^b^, 6 mL ^c^	hydrophilic–lipophilic balanced reversed-phase sorbent
CNW Poly-Sery MCX	92 μm, 500 mg, 6 mL	mixed-mode cation exchange sorbent
CNW Poly-Sery MAX	35–45 μm, 500 mg, 6 mL	mixed-mode anion exchange sorbent
CNW Poly-Sery XAD2	694 μm, 500 mg, 6 mL	non-ionic reticulated styrene–divinylbenzene polymer sorbent
CNWBOND LC-C18	40–63 μm, 500 mg, 6 mL	C18-bonded non-polar silica

^a^ Particle size; ^b^ sorbent bed weight; ^c^ SPE tube volume.

**Table 2:** The physicochemical properties and MS/MS conditions of 17 SAs.

Abbreviation	Full Name	Molecular Weight	Log *K*_OW_^a^	pKa ^b^	Use	Retention Time (min)	Precursor Ion (*m/z*)	Product Ion (*m/z*)	Cone Voltage (V)	Collision Energy (eV)
SG	sulfaguanidine	214.24	NA	NA	human	1.03	215	108, 156 *	34	20, 15
SG-D_4_		218.27				1.03	219	160 *	34	15
SP	sulfapyridine	249.29	0.35	2.90, 8.54	human and veterinary	3.12	250	156 *, 184	28	15, 16
SP-^13^C_6_		255.24				3.12	256	162 *	28	16
SD	sulfadiazine	250.28	−0.09	2.00, 6.48	human and veterinary	2.30	251	92, 156 *	23	27, 15
SD-^13^C_6_		256.24				2.30	257	162 *	23	15
ST	sulfathiazole	255.32	0.05	2.20, 7.24	human and veterinary	2.81	256	92, 156 *	26	25, 15
ST-D_4_		259.34				2.76	260	160 *	26	15
SM1	sulfamerazine	264.30	0.14	2.06, 6.90	human and veterinary	3.51	265	92, 156 *	24	25, 15
SM1-^13^C_6_		270.26				3.51	271	162 *	24	15
SML	sulfamethizole	270.33	0.54	1.86, 5.29	veterinary	5.42	271	92, 156 *	19	30, 15
SML-^13^C_6_		276.29				5.42	277	162 *	19	15
SMZ	sulfamethoxazole	253.28	0.89	1.85, 5.60	human and veterinary	9.28	254	92, 156 *	27	26, 16
SMZ-^13^C_6_		259.23				9.27	260	162 *	27	16
SIZ	sulfisoxazole	267.30	1.01	1.66, 4.71	human and veterinary	10.19	268	92, 156 *	22	28, 13
SIZ-^13^C_6_		273.26				10.19	274	162 *	22	13
SIM	sulfisomidine	278.33	NA	NA	human	1.96	279	156 *, 186	30	18, 25
SIM-D_4_		282.31				1.96	283	160 *	30	18
SM2	sulfamethazine	278.33	0.89	2.65, 7.65	human and veterinary	5.19	279.1	92, 186 *	30	28, 16
SM2-D_4_		282.35				5.11	283.1	186 *	30	16
SMM	sulfamonomethoxine	280.30	0.70	1.98, 5.96	human and veterinary	7.73	281	92 *, 156	28	31, 22
SMM-D_4_		284.33				7.63	285	96 *	28	22
SMP	sulfamethoxypyridazine	280.30	0.32	2.09, 6.95	human and veterinary	5.80	281	156 *, 215	34	20, 15
SMP-D_3_		283.32				5.69	284	156 *	34	20
SM	sulfameter	280.30	0.41	1.87, 6.50	human and veterinary	5.24	281	156 *, 215	32	20, 18
SM-D_4_		284.33				5.16	285	160 *	32	20
SDZ	sulfachloropyridazine	284.72	0.31	1.87, 5.45	human and veterinary	7.80	285.1	92, 156 *	22	28, 15
SDZ-^13^C_6_		290.68				7.80	291.1	162 *	22	15
SQ	sulfaquinoxaline	300.34	1.68	1.86, 5.56	veterinary	11.50	301.1	92.2, 156.1 *	26	30, 16
SQ-^13^C_6_		306.29				11.50	307.1	162.1 *	26	16
SDM	sulfadoxine	310.33	0.70	1.52, 6.01	human	9.50	311	92, 156 *	20	32, 15
SDM-D_3_		313.35				9.42	314	156 *	25	20
SPM	sulfadimethoxine	310.33	1.63	1.87, 5.86	veterinary	11.40	311.1	92, 156 *	20	32, 21
SPM-D_6_		316.37				11.33	317.1	162.1 *	25	20

NA: not available; * quantitative ion. ^a,b^ The data are from Li et al. [26].

**Table 3:** Analytical characteristics of the proposed method.

Analyte	ILIS	Linear Range (μg/L)	Regression Equation	*r* ^2^	LOD ^a^ (ng/L)	LOQ ^b^ (ng/L)	EFs	Precision, RSD (%, *n* = 5)	
								Intra-Day	Inter-Day
SG	SG-D_4_	0.05−100	y = 0.87x + 0.28	0.9999	0.05	0.15	1008	1.44	5.52
SP	SP-^13^C_6_	0.05−100	y = 0.93x + 0.21	0.9998	0.01	0.03	1007	1.96	3.54
SD	SD-^13^C_6_	0.05−100	y = 0.79x + 0.24	0.9995	0.01	0.03	990	2.64	4.06
ST	ST-D_4_	0.05−100	y = 0.84x + 0.21	0.9995	0.012	0.04	1033	2.39	3.83
SM1	SM1-^13^C_6_	0.05−100	y = 0.76x + 0.18	0.9994	0.01	0.03	1021	3.35	2.58
SML	SML-^13^C_6_	0.05−100	y = 0.79x + 0.16	0.9999	0.02	0.06	1021	3.05	3.73
SMZ	SMZ-^13^C_6_	0.05−100	y = 0.82x + 0.18	0.9997	0.01	0.03	988	2.58	3.26
SIZ	SIZ-^13^C_6_	0.05−100	y = 0.81x + 0.21	0.9994	0.01	0.03	1018	2.76	3.64
SIM	SIM-D_4_	0.05−100	y = 0.96x + 0.09	0.9996	0.04	0.12	1004	3.76	8.9
SM2	SM2-D_4_	0.05−100	y = 0.79x + 0.17	0.9997	0.01	0.03	982	2.57	7.94
SMM	SMM-D_4_	0.05−100	y = 0.74x + 0.17	0.9998	0.02	0.06	1019	2.64	3.56
SMP	SMP-D_3_	0.05−100	y = 0.70x + 0.19	0.9995	0.01	0.03	1019	3.54	2.08
SM	SM-D_4_	0.05−100	y = 0.76x + 0.27	0.9993	0.015	0.05	995	3.84	3.00
SDZ	SDZ-^13^C_6_	0.05−100	y = 0.82x + 0.25	0.9993	0.02	0.06	1006	2.34	3.61
SQ	SQ-^13^C_6_	0.05−100	y = 0.82x + 0.60	0.9994	0.02	0.06	987	2.59	3.62
SDM	SDM-D_3_	0.05−100	y = 1.18x + 0.68	0.9992	0.01	0.03	1007	1.86	2.67
SPM	SPM-D_6_	0.05−100	y = 1.54x + 0.40	0.9998	0.01	0.03	1019	1.87	2.44

^a^ LOD (S/N = 3); ^b^ LOQ (S/N = 10).

## Data Availability

Data will be made available on request.

## Supplementary Materials

The following supporting information can be downloaded at: https://www.mdpi.com/article/10.3390/molecules28124694/s1, Figure S1: Effect of eluent volume on extraction efficiency: 1.0 L of ultrapure water spiked with 20 ng/L SAs, pH 7.0 (n = 3); Figure S2: Chemical structures of the 17 SAs; Figure S3: Map of sampling sites in Sanmen Bay, East China Sea; Figure S4: Automatic cartridge-disk universal solid phase extraction system (LabTech, China); Figure S5: UPLC–MS/MS chromatograms of SAs standard at 20 μg/L; Table S1: Recoveries of real water samples spiked with SAs obtained by applying the proposed automated SPE UPLC-MS/MS method; Table S2: Comparison of different methods for the analysis of SAs in water; Table S3: Concentrations (ng/L) of SAs in river water and seawater samples (ng/L).
