# Supplementary material for: An Automated Solid-Phase Extraction–UPLC–MS/MS Method for Simultaneous Determination of Sulfonamide Antimicrobials in Environmental Water
Source: Molecules. 2023 Jun 11;28(12):4694. doi: 10.3390/molecules28124694 (PMC10304559; doi:10.3390/molecules28124694)
Supplement: Supplementary file 1 [file molecules-28-04694-s001.zip › molecules-2439495-supplementary.pdf]

SUPPORTING INFORMATION

Automated solid-phase extraction: A simple and robust method for the analysis of sulfonamide antimicrobials in environmental water samples

Mengyu Qi[1,2], Pengfei He[2], Hongmei Hu[2,3,*], Tongtong Zhang[1,2], Tiejun Li[2,*], Xiaoning Zhang[4], Yilin Qin[1], Yingjie Zhu[1,2], Yuanming Guo[2]

[1] Institute of Marine and Fisheries, Zhejiang Ocean University, Zhoushan 316021, China.

[2] Key Laboratory of Sustainable Utilization of Technology Research for Fisheries Resources of Zhejiang Province, Zhejiang Marine Fisheries Research Institute, Zhoushan 316021, China.

[3] Department of Chemistry, Zhejiang University, Hangzhou 310007, China

[4] State Key Laboratory of Resource Insects, College of Sericulture, Textile andBiomass Sciences, Southwest University, Chongqing 400715, China.

Corresponding Author

Hongmei Hu

E-mail: huhm@zju.edu.cn

Tiejun Li

E-mail: litiejun1982@126.com

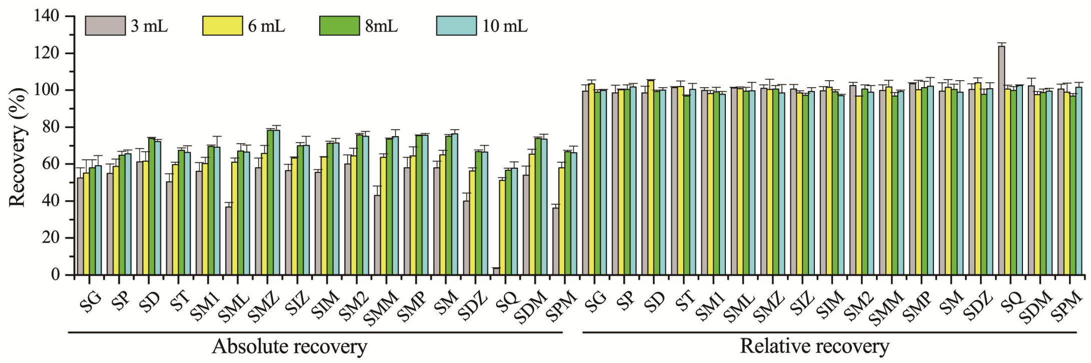

Figure S1. Effect of eluent volume on extraction efficiency: 1.0 L of ultrapure water spiked with 20 ng/L SAs, pH 7.0 (n=3).

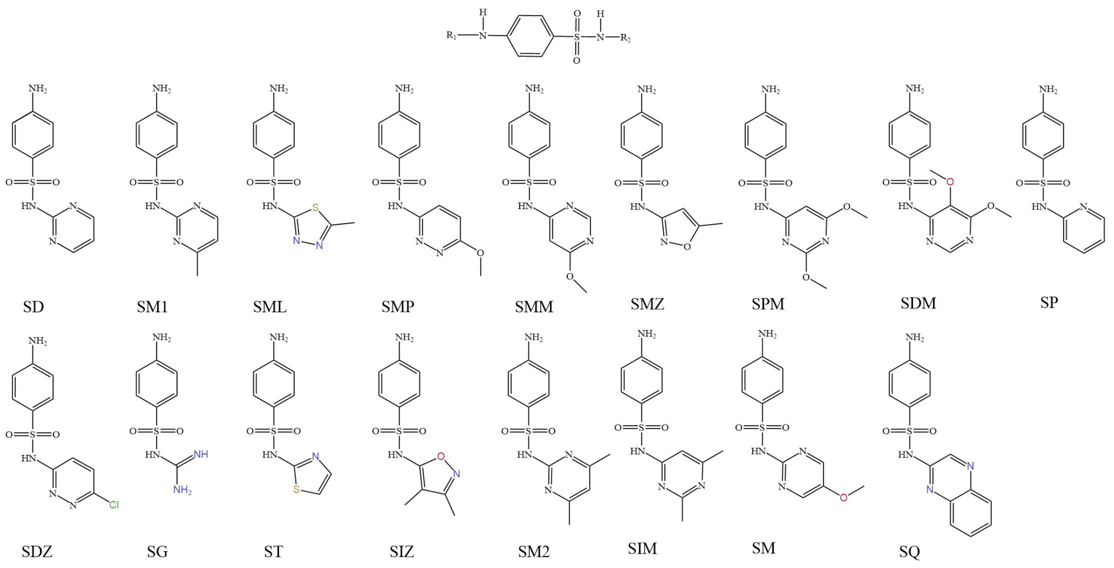

Figure S2. Chemical structures of the 17 SAs.

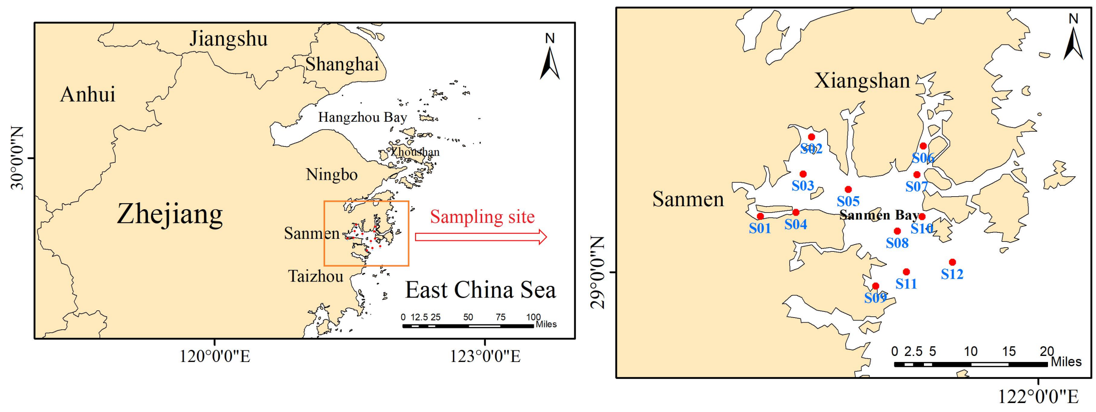

Figure S3. Map of sampling sites in Sanmen Bay, East China Sea.

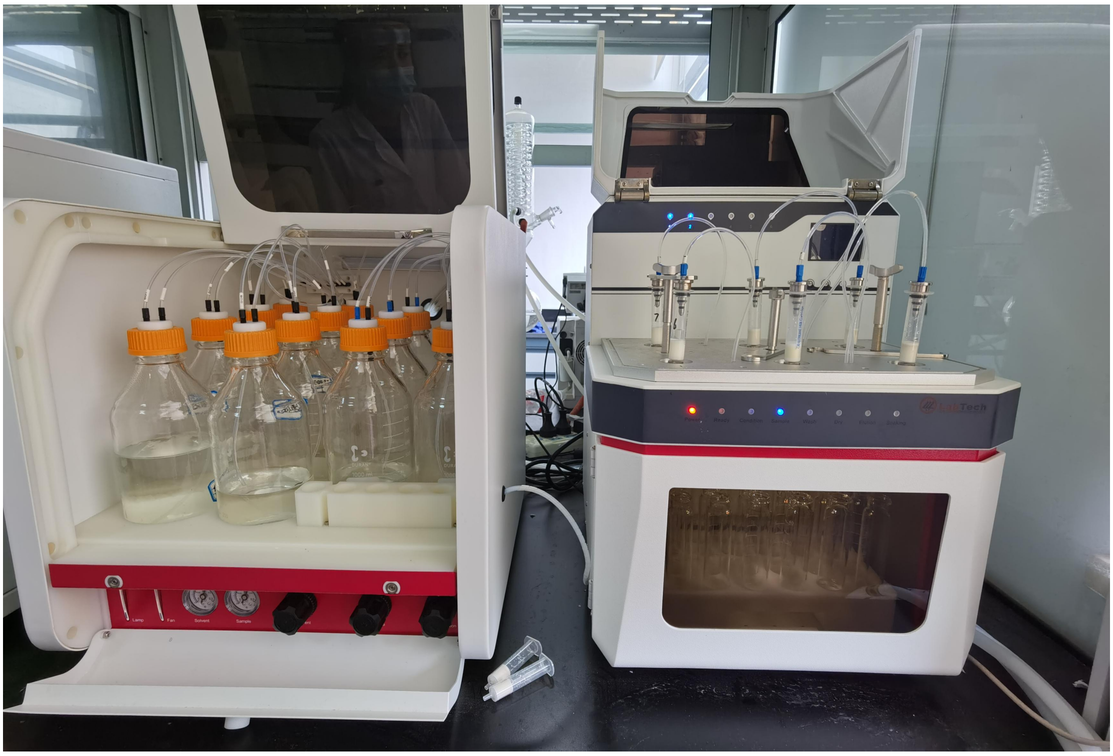

Figure S4. Automatic cartridge-disk universal solid phase extraction system (LabTech, China).

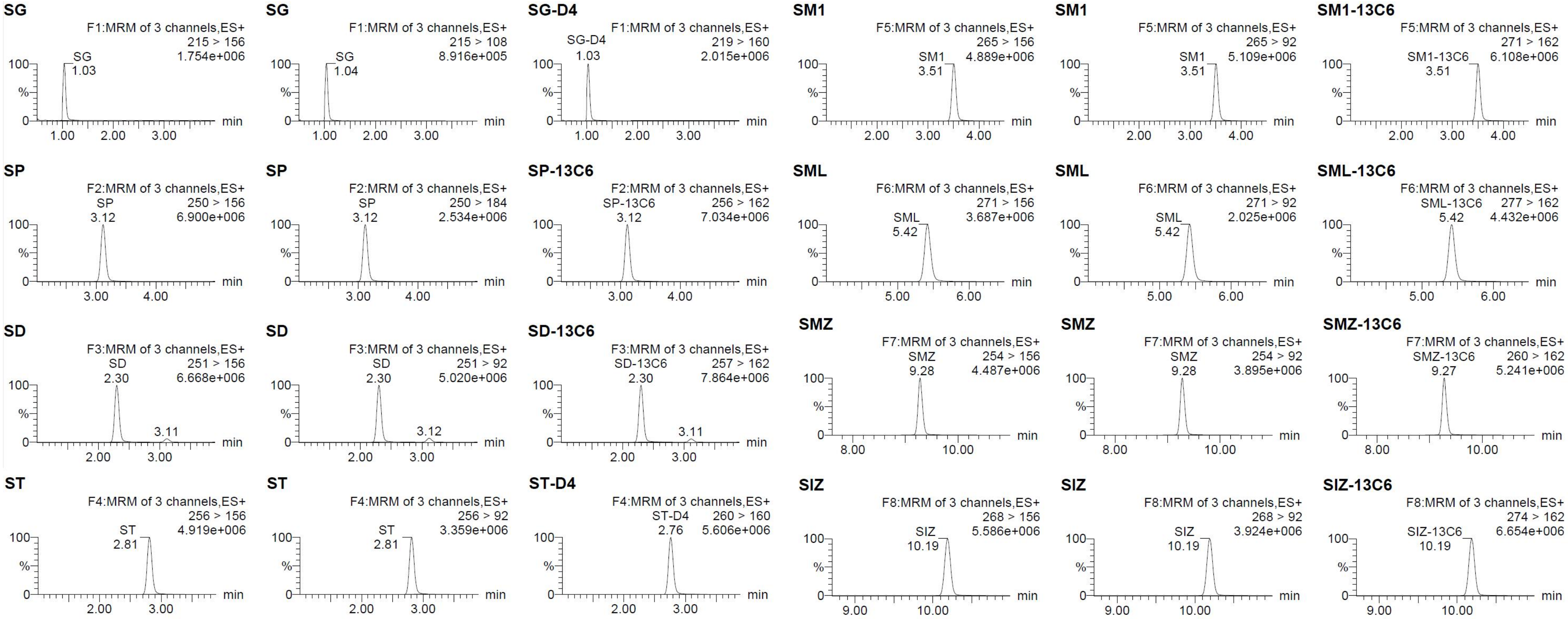

Figure S5-1. UPLC–MS/MS chromatograms of SAs standard at 20 µg/L.

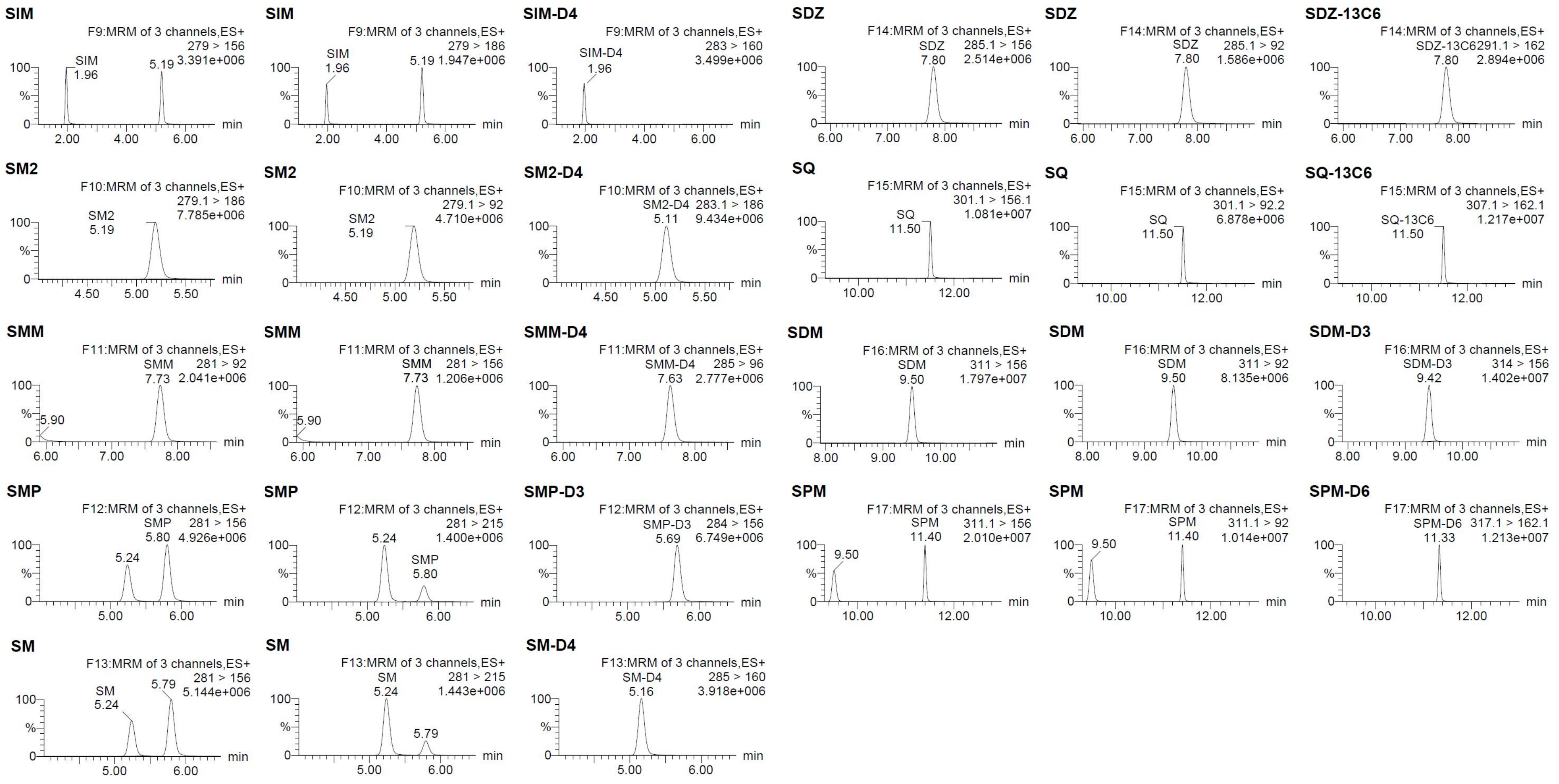

Figure S5-2. **UPLC–MS/MS chromatograms of SAs standard at 20 μg/L.**

Table S1. Recoveries of real water samples spiked with SAs obtained by applying the proposed automated SPE UPLC-MS/MS method.

Analyte	Pure water (n=5)		Tap water (n=5)		River water (n=5)		Seawater (n=5)	
	Spiked: 1 ng/L		Spiked: 10 ng/L		Spiked: 20 ng/L		Spiked: 100 ng/L	
	Recovery (%)	RSD (%)	Recovery (%)	RSD (%)	Recovery (%)	RSD (%)	Recovery (%)	RSD (%)
SG	93	5.9	102	2.4	90	6.9	111	0.4
SP	88	4.8	117	2.9	98	5.3	111	0.4
SD	105	8.6	111	3.1	102	10.0	112	5.1
ST	88	7.9	108	3.2	102	8.1	110	4.3
SM1	101	7.0	107	3.4	103	10.2	117	2.0
SML	99	1.2	106	1.4	101	10.7	116	2.0
SMZ	98	4.9	103	3.4	101	5.6	105	5.0
SIZ	99	1.8	107	3.2	100	9.8	113	2.8
SIM	86	8.8	109	5.4	96	5.0	104	1.0
SM2	82	5.9	109	0.9	91	5.6	116	2.6
SMM	102	6.9	110	2.8	103	9.2	114	0.3
SMP	88	1.6	103	3.5	104	8.2	118	2.2
SM	79	2.9	109	7.0	101	11.4	111	0.6
SDZ	98	14.5	104	4.4	100	10.0	116	1.5
SQ	102	14.2	105	3.2	103	8.2	105	2.4
SDM	88	10.2	101	1.6	102	9.4	117	0.7
SPM	100	12.9	108	2.0	97	9.1	113	1.7

Table S2. Comparison of different methods for the analysis of SAs in water.

Method	N*	Sample volume	Sample pretreatment	sorbent	Extract solvent	Elution or disperser solvent	Processing time (min)	LOD (ng/L)	Recovery (%)	RSD (%)	Ref.
manual SPE UPLC-MS/MS	18	1 L	Addition Na_2EDTA, pH adjustment of 2.0	200 mg of Oasis HLB	/	10 mL of 2% formic acid solution in methanol/acetonitrile(4:1, v/v)	> 250 min	0.014–0.293	84.8–108	<10	[15]
On-line SPE HPLC-MS/MS	16	5 mL	/	Oasis HLB	/	1 mL of acetonitrile, 1 mL of Methanol, and 1 mL of HPLC water	<20 min	0.03–3.3	39.7–134.4	<16	[12]
On-line SPE UPLC-MS/MS	9	10 mL	Addition formic acid	HyperSep Retain PEP[a]	/	methanol/acetonitrile(1:1, v/v)	20 min	1.32–7.91	65–169	<22	[16]
MIP-SPE HPLC-PDA	6	50 mL	pH adjustment of 7.0	20 mg of MIP(pre)[b]	/	1 mL of methanol	~21 min	10–14	87.4–102.3	<7	[10]
DLLME UPLC-DAD	11	5 mL	Addition NaCl, pH adjustment of 7.6	/	685 µL of chloroform	1250 µL of acetonitrile	<20 min	410–9870	78–117	≤20	[21]
MSPE UPLC-MS/MS	8	200 mL	pH adjustment of 4	15 mg of CMGO[c]	/	2.0 mL of methanol containing 1% (v/v) ammonia	~30 min	0.49–1.59	82–106.2	<7.2	[6]
In situ derivatization and HF LPME UPLC-FLD	8	8 mL	pH adjustment of 3.5	S 6/2 polypropylene hollow-fiber membrane	/	30 µL of pH 12.5 alkaline solution	~65 min	3.1–11.2	56–113	<20	[29]
In-tip SPME HPLC-PDA	3	10 mL	pH adjustment of 5–6	10 mg of activated charcoal	/	500 µL of 1% ammonium in methanol solution	<30 min	380~1140	82.8–108.7	<4.6	[11]
automated SPE UPLC-MS/MS	17	1 L	Addition Na_2EDTA, pH adjustment of 3.0	500 mg of CNW Poly-Sery HLB	/	8 mL of methanol : acetone (v/v, 1:1)	~60 min	0.01–0.05	79–117	<15	Present work

* Number of SAs evaluated.

[a] porous polystyrene divinylbenzene.

[b] multi-templates surface molecularly imprinted polymer with pre-polymerization process.

[c] carboxylated magnetic graphene oxide

Table S3. Concentrations (ng/L) of SAs in river water and seawater samples (ng/L).

		SG	SP	SD	SMZ	SM2	SMM	SDZ	∑SAs
River water (n=6)	Mean	2.35	4.787	1.936	8.072	0.256	0.304	ND	17.701
	Min	0.975	0.14	0.035	2.368	ND	ND	ND	8.157
	Max	4.823	10.033	6.898	14.446	0.556	0.691	ND	29.676
	DF(%)	100	100	100	100	83	67	0	100
Seawater (n=12)	Mean	0.506	0.723	1.361	9.833	0.22	4.213	0.128	16.984
	Min	ND	0.012	0.062	1.176	0.056	0.178	ND	1.683
	Max	1.733	1.825	2.176	27.605	0.47	19.867	0.423	36.955
	DF(%)	58	100	100	100	100	100	50	100

DF, detection frequency.

ND, not detected.

NC, not calculated, when the detection frequency <50%.
